# Social interaction effects: The impact of distributional preferences on risky choices

**DOI:** 10.1007/s11166-018-9275-5

**Published:** 2018-05-08

**Authors:** Anita Gantner, Rudolf Kerschbamer

**Affiliations:** Department of Economics, University of Innsbruck, Universitaetsstrasse 15, 6020 Innsbruck, Austria

**Keywords:** Peer effects, Social interaction effects, Social preferences, Risky choices, C91, D03, D63, D64, D81

## Abstract

This paper identifies convex distributional preferences as a possible cause for the empirical observation that agents belonging to the same group tend to behave similarly in risky environments. We first show theoretically that convex distributional preferences imply social interaction effects in risky choices in the sense that observing a peer choose a risky (safe) option increases the agent’s incentive to choose the risky (safe) option as well, even when lotteries are stochastically independent and the agent can only observe the lottery chosen by the peer but not the corresponding outcome. We then confirm our theoretical predictions experimentally.

**Electronic supplementary material:**

The online version of this article (10.1007/s11166-018-9275-5) contains supplementary material, which is available to authorized users.

## Introduction

People mostly act in social contexts rather than in isolation, and thus social comparison is typically part of an individual’s decision making process. A possible consequence of social comparison is that a decision maker (DM) makes his choice dependent on what he observes others in his reference group do. We shall refer to the latter as a *social interaction effect* if (i) the dependence is positive, i.e. if the DM’s propensity to choose a given activity is higher when (more) peers engage in the corresponding activity; and (ii) the dependence results from an increase in the DM’s utility payoff but not his material payoff when (more) peers engage in the corresponding activity.^1^ Social interaction effects have been invoked to explain correlations in risky choices in a great variety of different domains – for instance, in savings and investment decisions, employment choices, college entry and schooling decisions, substance use and criminal activity (see Scheinkman 2008 for a discussion of the literature).

Social comparison might not only affect decision making in risky environments, it is also a core element of some prominent models of *distributional preferences*.^2^ Preferences featuring inequality or inequity aversion (Fehr and Schmidt 1999; Bolton and Ockenfels 2000) have this property, as do maximin (Charness and Rabin 2002; Engelmann and Strobel 2004) or Leontief preferences (Andreoni and Miller 2002; Fisman et al. 2007) and envy (Bolton 1991; Kirchsteiger 1994; Mui 1995). Also, for altruism (Becker 1974; Andreoni and Miller 2002), surplus maximization (Engelmann and Strobel 2004), spite (Levine 1998) and concerns for relative income (Duesenberry 1949), which may be modeled without any reference to social comparison, there is ample empirical evidence indicating that social comparison influences behavior. For instance, Andreoni and Miller (2002) find that the choices of a large majority of givers in dictator games are consistent with convex altruistic preferences. Similarly, Kerschbamer (2015) reports that almost all subjects reveal (weakly) more benevolent (less malevolent) preferences in the domain of advantageous than in the domain of disadvantageous inequality – a pattern implied by convex distributional preferences. *Convexity* here refers to the property that a DM’s benevolence toward another individual increases (or that malevolence decreases) as the income of the other individual decreases along an indifference curve, and its strict incarnation obviously calls for social comparison.^3^

Convex distributional preferences have been invoked as potential explanation for non-standard behavior in important market and non-market environments – see Sobel (2005) and Fehr and Schmidt (2006) for excellent surveys of theoretical models and empirical evidence, and Cox et al. (2008) for an elegant theoretical investigation of the implications of convexity. The main focus of previous studies, however, has been on deterministic choices, while the effects of distributional preferences on behavior when choices are risky have found much less attention in the literature.

The novelty of this paper is to bring social interaction effects and distributional preferences together in a framework where the consequences of choices are risky and where lotteries are stochastically independent. More specifically, our main research question is whether and how the behavior of a DM with a concern for the material welfare of others is affected when risky choices are made in a context where the DM has the possibility to observe the choices of others in similar situations before making a decision. Our main theoretical result is that convex distributional preferences imply social interaction effects in risky choices. In particular, when a DM has convex distributional preferences and knows that a reference person (the “peer”) chooses a risky or safe option, following the peer’s choice increases the DM’s utility payoff even if his material payoff remains unaffected. The intuition for this result is that with convex distributional preferences an increase (decrease) in the final material payoff of a peer compared to the DM’s own final payoff increases (decreases) the relative weight the DM puts on own income. This introduces an asymmetry in the evaluation of unequal outcomes and thereby gives an incentive to behave similarly in risky environments.

We then test our predictions empirically. As pointed out by Manski (1993), a large part of the empirical literature on social interaction effects in risky choices is based on field data suffering from severe identification problems. We therefore set up our empirical investigation as a laboratory experiment, as such experiments allow for more control than other data sources. More specifically, since we are interested in the impact of information regarding a peer’s decisions on a DM’s choices in a risky environment without informational externalities and material payoff complementarities, the ideal data source would contain observations of the same DM’s choices in two such environments which differ only in the DM’s information regarding the action choice of the peer. While it seems almost impossible to get such data points in the field, in a lab experiment we can create an artificial environment that generates such points.

In our experiment, we investigate the choices of subjects in two risky environments that differ only in the information regarding the choices of a peer. We find large peer group effects in the aggregate data even though a subject’s decision has no impact on the peer’s monetary payoff, lotteries are stochastically independent, and the subject can only observe the lottery chosen by the peer but not the corresponding outcome. The problem of correctly identifying the relevant reference group of the subject is circumvented by providing only information about the behavior of a *single* peer. Since information externalities and material payoff complementarities are absent in the implemented environment, these potential sources for a positive correlation in the choices of the subject and the peer cannot explain our data. The fact that we observe the behavior of the *same* subject in two different environments in a within-subject design controls for self-selection and exogenous correlation of individual characteristics, and the fact that the two environments differ only in the information about the peer’s decisions excludes contextual and correlated effects as possible explanations. We therefore conclude that social interaction effects caused by convex distributional preferences are a plausible source for the observed correlation between the risky choices of DMs and their peers in the aggregate data.

To obtain further evidence in support of our hypothesis that social interaction effects are driven by convex distributional preferences, we also test our main predictions on the individual level. Using a non-parametric procedure to classify subjects regarding their distributional preferences, we find that social interaction effects are more pronounced for subjects with convex than for subjects with linear distributional preferences – which corresponds to the theoretical prediction. We also find some evidence in support of the theoretical prediction that the size of the social interaction effect is smaller for risk-neutral DMs than for risk-averse or risk-loving ones – as predicted by our model.

In risky environments, conformity has often been quoted as an explanation for differences between individual decision making and decisions within groups or with peers (see e.g. Bolton et al. 2015, or Lahno and Serra-Garcia 2015). As Cialdini and Goldstein (2004) put it, “conformity refers to the act of changing one’s behavior to match the responses of others.” Defined that way, conformity is not a motivation but rather an observed behavior based on some other underlying motivation. Thus, while what we observe might be called conformity, we provide an explanation for the observed behavior based on existing models of preferences. Specifically, in the theory part of the paper we show that existing models of social preferences imply a motive for conformist behavior when distributional preferences are convex; and in the experimental part we provide results that document social interaction effects in risky choices.

The rest of the paper is organized as follows. Section 2 discusses the related literature. Section 3 introduces the model and derives the theoretical results. Section 4 details the design, the predictions and the results of our experiment and compares the actual choices in the lab to the predicted behavior. Section 5 concludes. An Appendix in the Electronic Supplementary Material contains additional theoretical results and the experimental instructions.

## Related literature

In our discussion of the literature focusing on the interaction between social preferences and risk, we distinguish between situations where choices affect only the own material payoff of the DM, situations where choices affect the DM’s own payoff and possibly also the payoff of others, and situations where decisions are made entirely on behalf of others. The latter decisions are often called choices of impartial spectators, and the focus in the respective literature is on how the distribution of risk across different members of a society is perceived. Rohde and Rohde (2015) find that spectators are ex-ante inequality averse but ex-post inequality seeking in their choices of allocations of risk over groups of people. In Cettolin and Riedl (2016), the final outcome of spectators’ allocation decisions is uncertain, which induces large heterogeneity in justice views regarding the allocation of risk.

Turning to the literature investigating the choices of stakeholders that may affect others, Bolton and Ockenfels (2010) consider a context in which each DM chooses between a safe and a risky option, where the safe (risky) option implies a safe (risky) payoff for an anonymous recipient as well. The authors find that when the safe option yields inequality, the risky option is taken significantly more often, while the inequality resulting from the risky option does not affect risk taking. Similar in spirit are results reported by Güth et al. (2008) and by Brennan et al. (2008), who also investigate situations where DMs’ choices affect timing, risk or expected values of the payoffs of other agents.^4^

In contrast to the literature discussed thus far, in our model and in our experiments the choices of a DM have no effect on the material payoffs of other subjects. Specifically, we investigate a scenario where (i) lotteries of the DM and the peer are stochastically independent, (ii) the DM does not affect the material payoff of the peer and (iii) there is no information on the outcome of the peer’s lottery choice. We are aware of only one study investigating a constellation with those features: In Cooper and Rege (2011) subjects face individual gambles that differ in their ambiguity, and a subject’s choice has no impact on the material payoffs of other subjects. Different treatments control for a subject’s information regarding the choices of his peers. Cooper and Rege find large peer group effects in their aggregate data and present an explanation for these effects based on “social regret”, referring to a DM’s disutility when a non-chosen action would have led to higher payoffs ex-post, and where that regret is less intense if others have chosen the same action. Social regret then yields the result that observing a peer make a risky (safe) choice increases the incentive for the DM to choose the risky (safe) option as well. By contrast, we derive social interaction effects in a risky environment directly from existing models of distributional preferences and test the theoretical prediction both with aggregate data and on the individual level.

Somewhat less related to the present paper are the articles by Rohde and Rohde (2011), Bursztyn et al. (2014) and Lahno and Serra-Garcia (2015). Rohde and Rohde (2011) investigate whether one’s own risk attitude is affected by the risk others face. They find only little evidence that the risk exposure of others affects subjects’ own choice between risky alternatives, even though subjects showed concerns for inequality in a risk-free setting. The field experiment by Bursztyn et al. (2014) finds that social learning and social utility are important drivers for peer effects in financial decision making. Lahno and Serra-Garcia (2015) try to disentangle different channels for peer effects. Having the peer actively choose a lottery in one treatment while randomly assigning a lottery choice to the peer in another treatment allows them to distinguish between conformism and social preferences as possible explanations for the observed peer effects. Their results suggest that both channels contribute to the observed peer effects.

Other studies on risk taking in a social context investigate different research questions and environments: Corazzini and Greiner (2007) study whether inequality aversion can explain herding behavior in a social learning environment with common gambles; Linde and Sonnemans (2012) ask whether and how the payoff (rather than the decision) of a peer affects risk taking when the peer’s payoff is fixed either at a higher or a lower level than all possible lottery outcomes; Beckman et al. (2016) study how behavior toward risk is influenced by the position in the income distribution; Cettolin and Tausch (2015) apply the question of how a DM’s and a peer’s choice between risky options affects risk sharing, which implies redistribution, and Wakker et al. (2017) investigate how risky choices are affected by putting oneself in another person’s shoes.

## Theoretical model

Our workhorse model throughout the theory part of the paper is the piecewise linear utility or motivation function originally introduced by Fehr and Schmidt (1999) as a description of self-centered inequality aversion and later extended by Charness and Rabin (2002) to allow for other forms of distributional concerns. For simplicity, we concentrate on the case of two agents and two binary lotteries in the main text, deferring the more general case with more than two agents and more than two lotteries to Appendix A.1 in the Electronic Supplementary Material. For the two-agents case, the reciprocity-free version of the Charness and Rabin model reads

1 $$ u_{\rho,\sigma}(m,o)=\left\{ \begin{array}{c} m +\sigma (o-m)\hspace{0.5cm}for\hspace{0.5cm}o\geq m \\ m +\rho (o-m)\hspace{0.5cm}for\hspace{0.5cm}o<m \end{array} \right. \hspace{0.2cm}\forall \hspace{0.2cm}\sigma <1,\rho <1.  $$

where *m* (“my”) and *o* (“other”) stand for the material payoff of the DM and the peer, respectively. For the two parameters of the model we assume $\rho <1$ and $\sigma <1$ to guarantee strict monotonicity of utility in own material payoff.

Depending on the relation between the two parameters $\rho $ and $\sigma $ we distinguish between the following three cases:

(i) $\rho \!>\!\sigma $: indifference curves in the $(m,o)$-space that are convex; such preferences are thus referred to as *convex distributional preferences*;
(ii) $\rho =\sigma $: indifference curves in the $(m,o)$-space are linear; such preferences are thus referred to as *linear distributional preferences*
(iii) $\rho <\sigma $: indifference curves in the $(m,o)$-space are concave; such preferences are thus referred to as *concave distributional preferences*.

Convex indifference curves in the $(m,o)$-space imply that the DM is more benevolent (or less malevolent) in the domain of advantageous compared to the domain of disadvantageous inequality. Many well-known distributional preference models, such as inequity or inequality aversion, envy, maximin, Rawlsian or Leontief preferences, necessarily have this property, while some less prominent ones such as equity or equality aversion necessarily violate it.^5^ Altruism, surplus maximization and social welfare maximization, as well as spiteful or competitive preferences and concerns for relative income may or may not have this property.^6^

Suppose now a DM with preferences represented by the utility function (1) faces the choice between the two lotteries $L_{r}$ (“riskier”) and $L_{s}$ (“safer”). $L_{r}$ yields outcome $x_{r}$ with probability $p_{r}$ and zero with probability $1-p_{r}$, and $L_{s}$ yields outcome $x_{s}$ with probability $p_{s}$ and zero with probability $1-p_{s}$, where $x_{r}>x_{s}$ and $p_{r}<p_{s}$ (note that we allow for $p_{s}= 1)$. Throughout we assume that when both agents – DM and peer – choose the same lottery, each agent faces idiosyncratic risk, as our main research question is how the mere observation of the peer’s choice affects the DM’s decision between the two lotteries. We refer to a DM as risk-neutral if he prefers the lottery with the higher expected value, as risk-averse if he prefers the safer of two lotteries even for some range of lottery parameters where the risky lottery has the higher expected value, and as risk-loving if he prefers the riskier of two lotteries even for some range where the safer lottery has the higher expected value.

To calculate expected utilities, all possible final outcomes are evaluated and weighted by their respective probabilities. That is, in the sense of Fudenberg and Levine (2012), or Saito (2013), we assume that agents care for ex-post fairness in making their choices under risk. Fudenberg and Levine (2012) and Saito (2013) also discuss the alternative perspective where agents care for ex-ante fairness – that is, for fair lotteries instead of fair outcomes. Here it is important to note that the focus of these papers on social preferences under risk – as well as that of the most related experimental studies (see, e.g., Rohde and Rohde 2011, or Cettolin and Riedl 2016) – is on a DM who can sacrifice own payoff for the peer’s benefit, and vice versa. The question in this context then is – as Saito (2013) puts it – whether there is a preference for equality of opportunities or equality of outcomes. In our setting, each DM makes his own choice between independent lotteries, and a DM is not directly responsible for the outcome of another DM through his own choice. The only way the peer’s choice can affect the DM’s utility (and vice versa) is through a comparison of outcomes, since choices and lotteries are independent. In such a context we consider it to be unlikely that a DM’s choice is driven by a concern for ex-ante fairness. Rather, the DM’s concern about his own standing compared to that of a peer’s seems more likely to be shaped by a comparison of final outcomes.

Our first result summarizes how the DM’s risk attitudes are affected by social comparisons.

### **Proposition 1** (Distributional Preferences and Risk Attitudes with Risk-Neutrality in Isolation)

*Suppose the preferences of a DM can be represented by a utility function as* *defined in* Eq. 1*. Then the DM displays the following behavior in a social* *context:*

(i) *Given that the DM observes the peer choose* *lottery* $L_{r}$ *,* *he makes a risk-neutral choice independently of whether his distributional* *preferences are convex, linear, or concave.*
(ii) *Given that the DM observes the peer choose* *lottery* $L_{s}$ *,* *he makes a risk-averse choice if his distributional preferences are convex,* *a risk-neutral choice if his distributional preferences are linear, and a* *risk-loving choice if his distributional preferences are concave.*

### Proof

Let $u_{ln}$ denote the DM’s expected utility when he chooses lottery $L_{l}$, with $l=r,s$, while his peer chooses $L_{n}$, with $n=r,s$. That is, $u_{rr}$ denotes the DM’s expected utility when both agents choose $L_{r}$, $u_{rs}$ denotes the DM’s expected utility when the DM chooses $L_{r}$ while the peer chooses $L_{s}$, etc. Then we have

2 $$\begin{array}{@{}rcl@{}} u_{rr} & = & {p_{r}^{2}}x_{r} + p_{r}(1-p_{r})(x_{r}-\rho x_{r})+(1-p_{r})p_{r}\sigma x_{r}; \end{array} $$

3 $$\begin{array}{@{}rcl@{}} u_{rs} & = & p_{r}p_{s}[x_{r}-\rho(x_{r}-x_{s})]+p_{r}(1-p_{s})(x_{r}-\rho x_{r})+(1-p_{r})p_{s}\sigma x_{s}; \end{array} $$

4 $$\begin{array}{@{}rcl@{}} u_{sr} & = & p_{s}p_{r}[x_{s}+\sigma(x_{r}-x_{s})]+p_{s}(1-p_{r})(x_{s}-\rho x_{s})+(1-p_{s})p_{r}\sigma x_{r}; \end{array} $$

5 $$\begin{array}{@{}rcl@{}} u_{ss} & = & {p_{s}^{2}}x_{s}+p_{s}(1-p_{s})(x_{s}-\rho x_{s})+(1-p_{s})p_{s}\sigma x_{s}. \end{array} $$

For (i), suppose the peer chooses the riskier lottery $L_{r}$. Then the DM prefers $L_{s}$ to $L_{r}$ if and only if $u_{sr}>u_{rr}$, which simplifies to

6 $$\begin{array}{@{}rcl@{}} p_{s}x_{s}[1-\rho+p_{r}(\rho-\sigma)] & > & p_{r}x_{r}[1-\rho+p_{r}(\rho-\sigma)]. \end{array} $$

It is now straightforward to verify that independent of whether the DM has convex, linear, or concave distributional preferences the term in brackets is strictly positive. For convex distributional preferences this follows directly from $\rho <1$ and $\sigma <\rho $, for the linear case it follows from $\rho <1$ and $\sigma =\rho $. For concave distributional preferences note that $1-\rho +p_{r}(\rho -\sigma )>0$ can be restated as $1-\sigma p_{r}>\rho (1-p_{r})$, and that the LHS of the latter condition is decreasing in $\sigma $ and has its infimum at $1-p_{r}$, while the RHS is increasing in $\rho $ and has its supremum at $1-p_{r}$. Thus, for all three considered cases of distributional preferences, condition (6) is equivalent to $p_{s}x_{s}>p_{r}x_{r}$, implying that if the peer chooses the riskier lottery, the DM chooses the lottery with the higher expected value, i.e. his choice is independent of the distributional parameters $\sigma $ and $\rho $.

For (ii), suppose that the peer chooses the safer lottery $L_{s}$. Then the DM prefers $L_{s}$ over $L_{r}$ if and only if $u_{ss}>u_{rs}$, which simplifies to

7 $$\begin{array}{@{}rcl@{}} \frac{\rho-\sigma}{1-\rho}& > & \frac{p_{r}x_{r}-p_{s}x_{s}}{p_{s}x_{s}(p_{s}-p_{r})}. \end{array} $$

For a DM with convex distributional preferences the LHS is strictly positive since $\sigma <\rho <1$, for a DM with linear distributional preferences the LHS is zero since $\sigma =\rho <1$, and for a DM with concave distributional preferences the LHS is strictly negative since $\rho <\sigma <1$. Thus, as long as the safer lottery has the higher expected value, a DM with convex distributional preferences prefers it. He may prefer the safer lottery even when it has a lower expected value, as long as the expected value is not too much lower than that of the riskier lottery, where the exact condition is given in Eq. 7. According to our definition of risk attitudes above, he thus makes a risk-averse choice in a social context, even though he was assumed to be risk-neutral in isolation. The argument for the other two cases is similar. □

Proposition 1 shows that social comparisons affect the risky choices of a DM with non-linear distributional preferences even when the DM is an expected-value maximizer when acting in isolation. Social information can thus be an independent source driving risky choices. Proposition 1 has important implications. An immediate one is that the well-known inequality aversion model of Fehr and Schmidt (1999) implies risk-averse behavior in a social environment when the peer chooses the safer of two lotteries, even though risk neutrality is assumed when acting in isolation. The same is true for the quasi-maximin model of Charness and Rabin (2002) and for many other distributional preference models in use in experimental economics and beyond. This is an important insight because it might help explain why subjects in the lab tend to behave in a risk-averse manner despite the low stakes involved. Since the overwhelming majority of DMs who are not exclusively interested in the maximization of their own material income has convex distributional preferences (for experimental evidence see, e.g., Fehr and Schmidt 1999; Andreoni and Miller 2002; Fisman et al. 2007), Proposition 1 predicts risk-averse behavior, on average, even if all subjects would behave in a risk-neutral manner in isolation.

Our next result summarizes the impact of the peer’s behavior on the choices of the DM in a risky environment. When comparing risky choices of a DM in different environments, it is useful to consider the DM’s indifference point between the lotteries, i.e. the probabilities $p_{r}$ and $p_{s}$ at which the DM is just indifferent between $L_{r}$ and $L_{s}$. If information regarding the choice of a peer has any effect, this indifference point must change. In other words, rather than being indifferent between lotteries, the new information induces the DM to have a strict preference for one of the lotteries.

### **Proposition 2** (Distributional Preferences and Social Interaction Effect with Risk-Neutrality in Isolation)

*Suppose a DM whose preferences can be represented* *by a utility function as in* Eq. 1 *is indifferent between* *lotteries *$L_{r}$ *and *$L_{s}$ *when* *he observes the peer* *choose *$L_{s}$ *(L*_*r*_*).* *Then observing the peer* *choose *$L_{r}$ *(L*_*s*_*)* *instead induces the following behavior: If the DM’s distributional preferences* *are*

(i) *convex then he chooses* $L_{r}$ *(* *L*_*s*_ *);*
(ii) *linear then he is indifferent between the two lotteries and chooses* *either* $L_{r}$ *or* $ L_{s}$ *;*
(iii) *concave then he chooses* $L_{s}$ *(* *L*_*r*_ *).*

### Proof

For (i), we have to show that for a DM with convex distributional preferences we have

- $u_{rr}=u_{sr}\Rightarrow u_{rs}<u_{ss}$, and
- $u_{rs}=u_{ss}\Rightarrow u_{rr}>u_{sr}$.

Consider part (a): Recall from Proposition 1 that in a social context the DM makes risk-neutral choices if the peer chooses $L_{r}$. Thus, we can have $u_{rr} = u_{sr}$ if and only if $p_{s}x_{s} = p_{r}x_{r}$. Then, using Eqs. 3 and 5, we have

8 $$ u_{rs}<u_{ss} \iff p_{r}x_{r}(1-\rho)< p_{s}x_{s}[1-\rho+(p_{s}-p_{r})(\rho-\sigma)], $$

which is satisfied since $\rho >\sigma $ by convexity, $\rho <1$ by monotonicity, and $p_{r}x_{r}=p_{s}x_{s}$ by risk-neutrality of the DM. Now consider part (b): Recall from Proposition 1 that in a social context a DM with convex distributional preferences makes risk-averse choices if the peer chooses $L_{s}$. Thus, we can have $u_{rs} = u_{ss}$ only if $p_{r}x_{r} > p_{s}x_{s}$. Then, using Eqs. 2 and 4, we have

9 $$ u_{rr}>u_{sr} \iff p_{r}x_{r}[1-\rho +p_{r}(\rho-\sigma)]+p_{r}x_{r}\sigma>p_{s}x_{s}[1-\rho +p_{r}(\rho-\sigma)]+p_{r}x_{r}\sigma, $$

which is satisfied since $\rho >\sigma $ by convexity, $\rho <1$ by monotonicity, and $p_{r}x_{r} > p_{s}x_{s}$ by risk-aversion of the DM. The proofs for (ii) and (iii) follow similar lines. □

### Remark

Proposition 2 extends to the case of common gambles, i.e. when the lottery outcomes are perfectly correlated across agents. We show this in Appendix A.2 in the Electronic Supplementary Material.

Proposition 2 contains our main theoretical result regarding social interaction effects. It states that convex distributional preferences imply social interaction effects in risky choices in the sense that observing a peer choose a risky (safe) option increases the agent’s incentive to choose the risky (safe) option as well, even when lotteries are stochastically independent and the agent can only observe the lottery chosen by the peer but not the corresponding outcome. This is a result with important empirical implications if one takes into account the ample existing evidence for convex distributional preferences.

So far, we have only considered the case where agents are risk-neutral in isolation. We now allow for more general risk attitudes in isolation by assuming that the DM values his own material payoff with the von-Neumann-Morgenstern utility function $v(m)$. In this context, risk-averse (risk-loving) preferences are captured by the concavity (convexity) of $v(m)$. Appendix B in the Electronic Supplementary Material describes in detail how Proposition 2 extends to the case where the DM has non-linear risk attitudes in isolation. Assuming that in a social comparison context the DM is risk-neutral regarding the difference between his own and his peer’s payoff, but may display other risk attitudes regarding his own material payoff, the extension of Proposition 2 (Proposition A3 in Appendix B) states that for an agent with convex distributional preferences, observing a peer choose a risky (safe) option increases the agent’s incentive to follow the peer’s choice. Analogously, a DM with concave distributional preferences has the tendency to deviate from the peer’s behavior. We also show that the impact of the peer’s behavior on the DM’s choice in a risky environment is larger for a DM who is not risk-neutral in isolation. The DM does not need to know the peer’s risk preferences, as he only relies on the peer’s actual choices, irrespective of the motivation behind the choices. Next we test these predictions in an experimental setting.

## Experiment

### Experimental setup

Our experimental design features three treatments which differ in the information provided to subjects regarding a peer’s choices in a risky environment. Within each of the three treatments, there are two distinct parts: In Part 1 we elicit subjects’ distributional preferences using a non-parametric elicitation procedure. In Part 2 subjects are exposed to 30 binary choices between a sure payoff and a lottery. We first describe the decision tasks in the two parts (which are identical across treatments), then the treatments (differing in the information subjects receive in Part 2 of the experiment), and finally the experimental procedures.

#### Decisions in Part 1

We follow the Equality Equivalence Test introduced by Kerschbamer (2015). In this procedure, each subject is exposed to a series of choices between two allocations, each specifying a payoff for the subject (“the DM”) and one for an anonymous partner (“the passive agent”). In each choice task, one of the two allocations is symmetric (that is, it involves equal material payoffs) while the other allocation is asymmetric. In one half of the choice tasks the asymmetric allocation is such that the DM is ahead, in the other half the asymmetric allocation is such that the DM is behind. The former choice tasks are labeled as “Advantageous Inequality Block” in Table 1, the latter as “Disadvantageous Inequality Block”.

**Table 1:** Test for distributional preferences: Paired choices

Disadvantageous inequality block				Advantageous inequality block			
Left		Right		Left		Right	
You	Other	You	Other	You	Other	You	Other
15	30	20	20	15	10	20	20
19	30	20	20	19	10	20	20
20	30	20	20	20	10	20	20
21	30	20	20	21	10	20	20
25	30	20	20	25	10	20	20

We used the parametrization of the procedure displayed in Table 1, with an exchange rate of 0.10 Euro per Experimental Currency Unit (ECU). When making their choices, subjects knew that (i) their earnings for this part of the experiment would be determined at the end of the experiment; (ii) they would receive two cash payments for this task, one as a DM and one as a passive agent; (iii) for their earnings as a DM one of the 10 decision problems would be selected by a random draw made separately for each participant and the alternative chosen in this decision problem would be paid out; and (iv) their earnings as a passive agent would come from another participant (i.e., not from the passive agent of the subject under consideration).

As shown by Kerschbamer (2015), in each of the decision blocks a rational DM switches at most once from the symmetric to the asymmetric allocation, and the switch points in the two blocks are informative about the DM’s type and intensity of distributional preferences. Moreover, they can also be used to obtain estimates of the two parameters $\rho $ and $\sigma $ of the functional form (1).^7^ This is the information we are interested in, and we use this information to classify subjects as having either convex (*ρ* > *σ*), linear (*ρ* = *σ*), or concave (*ρ* < *σ*) distributional preferences.

#### Decisions in Part 2

Here, subjects were exposed to a series of 30 binary choices between a cash gamble and a sure payoff. The binary choices were shown one by one on the screen, and in a given decision round all participants faced the same pair of alternatives. Participants knew that in each decision round they would have either an active or a passive role. A pair of alternatives could show up more than once, and in case it appeared again, subjects in an active role had to make a new decision, while for subjects in a passive role the computer would automatically implement the decision they made the first time they saw this pair of alternatives.^8^ In each decision round, subjects were privately informed about whether they were in an active or passive role. In some decision rounds, participants in an active role were also informed about the decision of a subject in the passive role.^9^ Actual earnings of a subject were determined at the end of the experiment and depended on the realization of two separate random variables – one was session-specific, determining which of the 30 decision rounds would be payoff-relevant for all subjects in that session, and the other was subject-specific, determining the personal lucky number for the subject under consideration in case this subject chose the lottery in the payoff-relevant pair.^10^

Subjects were informed about all this at the beginning. They were made aware that each participant’s decision had consequences for his own earnings only. The instructions pointed out that a subject in the active role who is informed about the past decision of an (anonymous) peer knows precisely how this peer decides in the current round.^11^ They were also made aware that the two random draws for the determination of the earnings ensured that subject and peer receive their earnings from the same decision task and that in case both subjects decided for the risky option in that task, the realizations of the risky option are stochastically independent. The 30 decision tasks (which are the same for all subjects and in all treatments) are then presented in 3 blocks.

Block 1 contained the 10 choices between a sure payoff and a lottery displayed in Table 2. Choices were presented in an ordered sequence (a screen shot is provided in Appendix C in the Electronic Supplementary Material). Since the sure payoff was always 50 ECUs while the lottery yielded 100 ECUs with probability *p* and 0 ECU with probability $1-p$, and since the probability *p* increased from one pair to the next, a rational DM switches at most once from the sure payoff to the cash gamble (and never in the other direction) and the switch point is informative about the DM’s risk attitude.^12^ For simplicity, we will use the number of safe choices in Block 1 as a proxy for a subject’s indifference probability in isolation.^13^ Since a risk-neutral subject would be indifferent between the sure payoff and the cash gamble for pair 3 in Table 2, subjects who make two or three safe choices are classified as risk-neutral, subjects who make at most one safe choice are classified as risk-loving and subjects who make at least four safe choices are defined as risk-averse. In Block 2 and Block 3 subjects faced the same 10 paired choices as in Block 1, now with the additional information on their own previous decision for the corresponding pair in Block 1 and in some treatments also with information about a peer’s decision (as explained below).

**Table 2:** Test for risk preference: Paired choices

Pair No.	Sure payoff	Lottery
1	50	100 with $p = 0.40$, 0 with $1-p$
2	50	100 with $p = 0.45$, 0 with $1-p$
3	50	100 with $p = 0.50$, 0 with $1-p$
4	50	100 with $p = 0.55$, 0 with $1-p$
5	50	100 with $p = 0.60$, 0 with $1-p$
6	50	100 with $p = 0.65$, 0 with $1-p$
7	50	100 with $p = 0.70$, 0 with $1-p$
8	50	100 with $p = 0.75$, 0 with $1-p$
9	50	100 with $p = 0.80$, 0 with $1-p$
10	50	100 with $p = 0.85$, 0 with $1-p$

#### Treatments

Our experimental design features three treatments: In treatments RLF (Risk Loving First) and RAF (Risk Averse First) information about the choices of a peer was presented to subjects in an active role, while in treatment NOP (No Peer) peer information was absent. In each session of a treatment with peer information, the computer program identified the most risk-loving and the most risk-averse subject from the decisions in Block 1. Each of these two subjects was in the passive role in one of the following two blocks, while all other subjects were in the active role. A subject in the passive role in a given block served as the peer for the other subjects in that block. In RLF, the most risk-loving subject in Block 1 served as the peer in Block 2, and the most risk-averse subject in Block 1 served as the peer in Block 3. In treatment RAF, this order was reversed – that is, the most risk-averse subject in Block 1 served as the peer in Block 2, and the most risk-loving subject in Block 1 served as the peer in Block 3.^14^ By comparing the choices a subject in the active role made in Block 2 to those he made in Block 3, we address the question of whether the peer’s choice affected the decisions of the subject under consideration, as the choices in the two blocks differ only in the information about the peer’s decisions. In treatment NOP, where peer information was absent, subjects faced the same 10 paired choices (as displayed in Table 2) in the three blocks, with information in Block 2 and Block 3 only about their own past choice in Block 1.

#### Procedures

The experiment was computer-based, using the software z-Tree (Fischbacher 2007). It consisted of 9 sessions conducted at the Innsbruck-Econ-Lab. A total of 166 subjects were recruited among undergraduate students of any major at the University of Innsbruck in May 2012 via the software ORSEE (Greiner 2015). Fifty-five subjects participated in RLF, 55 in RAF and 56 in NOP. Since we conducted 3 sessions for each treatment, and since each session except those for NOP included 2 peers, we remain with 49 subjects in the active role in treatments RLF and RAF, and 56 subjects in NOP, which gives a total of 154 subjects whose decisions will be analyzed below. Upon arrival, the instructions of Part 1 (identical for all subjects across all treatments) were read aloud to ensure common knowledge. Subjects then had time to read the instructions in private and to ask questions. After Part 1 was completed, instructions for Part 2 (identical for all subjects of a session) were distributed and read aloud, and subjects again had time to read them in private and to ask questions. At the end of each session a bingo cage with numbered balls was used for the random draws in the lab, and all draws could be followed by all participating subjects of a session. Sessions lasted about 45 minutes and participants averaged earnings of 10.30 Euro.

### Experimental predictions

Our main hypothesis for the aggregate data is motivated by the empirical evidence gathered by psychologists and experimental economists in the last decades showing that (i) distributional preferences are behaviorally relevant in many contexts, and (ii) the overwhelming majority of subjects who are not exclusively interested in the maximization of their own material income have convex distributional preferences. According to our theoretical results, convex distributional preferences imply that observing the peer choose a risky (safe) option increases the DM’s propensity to choose the risky (safe) option as well, even when lotteries are stochastically independent and the agent can only observe the lottery chosen by the peer but not the corresponding outcome. Our main prediction for the aggregate data is therefore:

#### **Prediction 1** (**Social Interaction Effect in Aggregate Data**)

*For two decision blocks with identical ordered pairs of lottery choice options* *for the DM and the peer, but different actual choices of the peer, subjects on* *average follow the behavior of the peer.*

We will test Prediction 1 by comparing the number of safe choices in Block 2 to the corresponding number in Block 3.^15^ For treatment RLF, evidence indicating that this number is lower in Block 2 than in Block 3 is interpreted as evidence in support of the prediction, as is evidence in RAF indicating that this number is higher in Block 2 than in Block 3. An alternative way to test Prediction 1 is to ask – for those subjects who change their behavior between Block 2 and Block 3 – in which direction they change their behavior. If convex distributional preferences are the main driver for the changes in behavior, then more subjects should adjust their behavior in the direction of the peer than in the opposite direction, and we will search for evidence in support of this prediction.

The next two predictions look at the individual level. First, individual data should confirm that subjects classified as having convex distributional preferences are more likely to change their behavior in the direction of the peer than subjects with linear distributional preferences (which include material payoff maximizers).

#### **Prediction 2** (**Social Interaction Effect at the Individual Level**)

*For two decision blocks with identical ordered pairs of lottery choice options* *for the DM and the peer, but different actual choices of the peer, subjects with* *convex distributional preferences have a more pronounced tendency to follow* *the behavior of the peer than other subjects.*

We will test Prediction 2 by comparing the changes in the number of safe choices from Block 2 to Block 3 of subjects classified as having convex distributional preferences to those of subjects classified as having linear distributional preferences.^16^

Our last prediction regards the extension of Proposition 2, which showed that for any combination of the distributional preference parameters $\rho $ and $\sigma $ the impact of the peer’s choice is less pronounced for risk-neutral DMs than for risk-loving and risk-averse agents (see Proposition A3 in Appendix B).

#### **Prediction 3** (**Risk Preferences and Social Interaction Effect**)

*For two decision blocks with identical ordered pairs of lottery choice options* *for the DM and the peer, but different actual choices of the peer, risk-neutral* *subjects have a less pronounced tendency to follow the behavior of the peer than* *risk-loving or risk-averse subjects.*

We will test Prediction 3 by comparing the changes in the number of safe choices from Block 2 to Block 3 of subjects classified as having risk-neutral preferences to those of subjects classified as having either risk-loving or risk-averse preferences.

### Experimental results

We start by looking at the aggregate data. Table 3 displays summary statistics for the number of safe choices in Blocks 1, 2 and 3, denoted as *b1safe, b2safe* and *b3safe*, for each of the three treatments. A comparison of *b1safe* across treatments shows no significant differences across populations (Kruskal-Wallis test: $p = 0.71$), indicating that the random assignment of subjects to the three treatments was successful. Since our main interest is the impact of a change in the peer’s choice on subjects’ decisions, we define the variable *change* $=$ *b2safe-b3safe* as a measure of the relevant shift in the indifference probability. Recall that in treatment RLF the peer in Block 2 makes fewer safe choices compared to the peer in Block 3. Thus, if subjects follow the peer’s choice on average, then *change* should be negative. This is exactly what we find in the data – see Table 3. By contrast, in RAF the peer in Block 2 makes more safe choices compared to the peer in Block 3, and thus following the peer would imply that *change* is now positive, which is again what we observe, on average. On the aggregate, we thus find that irrespective of the order in which peer choices are presented (RAF vs. RLF), subjects’ behavior follows peers’ behavior. Instead, our treatment without any peer (NOP), while also showing some change from Block 1 to Block 2, does not display a significant change from Block 2 to Block 3: The t-tests in Table 3 indicate that only in the two treatments with peers is *change* significantly different from zero and has the expected sign.

**Table 3:** Number of safe choices by block and treatment

	Treatment RLF			Treatment RAF			Treatment NOP		
	*b1safe*	*b2safe*	*b3safe*	*b1safe*	*b2safe*	*b3safe*	*b1safe*	*b2safe*	*b3safe*
Mean	4.51	3.96	4.61	4.77	5.58	5.10	5.08	5.61	5.5
Median	4	3	4	4	6	5	4.5	6	5.5
Std.Dev.	2.64	2.31	2.40	2.99	2.89	2.91	3.27	2.77	2.74
*change*	− 0.65			0.47			0.11		
t-test	*p* < 0.01			*p* < 0.03			*p* = 0.38		

We define *change* $=$ *b2safe-b3safe*

**Fig. 1:**
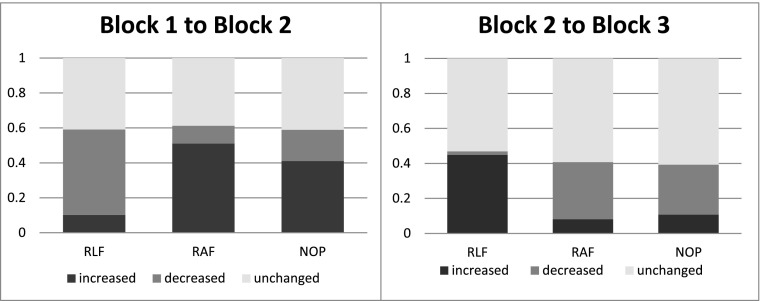
Change in fraction of safe choices over blocks

Turning to within-subject comparisons, Fig. 1 displays the proportion of subjects for whom the number of safe choices changed when moving from one block to the next. Again, our main focus is on the change in behavior when moving from Block 2 to Block 3.^17^ Here, in RLF we find that $47\%$ of subjects change the number of safe choices; all but one of these subjects change their behavior into the peer direction; in RAF $41\%$ change their behavior, and $80\%$ of those who change move into the peer’s direction. Finally, we observe that in NOP $39\%$ of subjects also change their behavior, and that $73\%$ of those who change move to more risk-loving choices in Block 3, even though they do not have any information except their own past choices. The Wilcoxon Signed Rank (WSR) test confirms that our proxy for the indifference probability shifts into the predicted direction in the treatments with a peer (*p* < 0.01 for both, RLF and RAF). However, our treatment NOP without peer also displays a significant shift in behavior from Block 2 to Block 3 toward more risky choices (WSR: $p<0.07$). That is, subjects in NOP seem to display a change in behavior that is qualitatively similar to that in RAF. The shift in NOP, however, cannot be based on any exogenous factor, since subjects face identical decisions and identical information as in the previous block.

Since we observe changes in behavior for all three treatments, we test whether the change of subjects’ decisions in treatments RLF and RAF can really be attributed to the information about the peer’s choice. To address this question, we define the binary variable *choice* taking the value 1 if a subject chose the sure payoff and 0 if the subject chose the lottery in a given decision. Table 4 shows the results of a logit regression, where the dependent variable *choice* for Block 2 and Block 3 is explained by *b1choice* – representing a subject’s own past choice in Block 1 – and by the influence of the peer. In this regression, we treat the observed choices in NOP *as if* the same peer information was present as in RAF, i.e. the variable *peerRA*, representing an indicator variable for a risk-averse peer, is set to 1 in Block 2 of RAF and NOP, while it is set to 1 in Block 3 of RLF.

**Table 4:** Logit regression of individual choices

Regressor	Coefficient	(Rob. SE)	$P>z$
*b1choice*	4.410	(0.203)	0.000
*peerRA*	0.113	(0.131)	0.387
*peer*	$-$ 0.678	(0.250)	0.007
peer*peerRA	0.504	(0.186)	0.007
*cons*	$-$ 1.732	(0.217)	0.000

Logit regression for *choice*; N $=$ 3080; Std. Err. adjusted for 154 clusters;

*Prob* > *χ*^2^ = 0.000; Pseudo $R^{2} = 0.5247$

The variable *peer* indicates whether or not there was a peer present, and the cross-term peerRA*peer should then give an indication of whether the choices in NOP can be explained in the same way as the choices in RAF. In line with theory and experimental design, the results displayed in Table 4 show that the choices in RAF and those in NOP are not explained by the same model: While the coefficient for *peerRA* is not significant, the coefficient for the cross-term peerRA*peer is highly significant. That is, only if there is a real peer and the peer decides in a risk-averse manner does this increase the probability of making the safe choice.^18^ We summarize our results based on aggregate data as follows:

#### **Result 1** (**Social Interaction Effect in Aggregate Data**)

*In line with Prediction* *1, subjects in treatments RLF and RAF follow, on* *average, the behavior of the peer.*

One might try to explain Result 1 by arguing that social information helps subjects operationalize their risk attitudes. While they are unsure what to prefer initially, they know better what to do when they receive information about the peer’s choice. This would be in line with social learning theory (see Bandura 1977) and with findings from social psychology (see, e.g., Yechiam et al. 2008). In our setting, however, it is not known whether following the peer’s choice leads to a better decision, since the consequences of his choices are not known. A low number of safe choices within a block might be attributed to the peer’s risk-loving attitude, but such information is not necessarily helpful for the DM’s own evaluation of the prospects, given his own risk attitude. It therefore seems rather unlikey that social learning is the main driver behind Result 1. More importantly, a learning story is hard to bring in line with the correlations on the individual level we report next.

To test Prediction 2 on the individual level, we distinguish between two classes of distributional preferences: convex preferences (*ρ* > *σ*) and linear preferences (*ρ* = *σ*). While 64 of 154 subjects (42*%*) fall into the former class, 68 (44*%*) are found in the latter, implying that we cover $86\%$ of all subjects with this classification. If we now compare our proxy for the changes in the indifference probability (*change*) across the two classes, we find that in treatments RLF and RAF $53\%$ of subjects with convex distributional preferences change behavior with changing peer information, while the corresponding fraction for linear types is only $25\%$. Pooled data of the two treatments shows that this difference is significant (*χ*^2^-test: $p<0.05$). By contrast, this explanation fails in treatment NOP, as subjects with convex distributional preferences are not more likely to change behavior: $75\%$ of convex types display unchanged behavior when moving from Block 2 to Block 3, while $47\%$ of linear types display a change in the number of safe choices (*χ*^2^-test: $p = 0.16$). This supports our hypothesis that the social interaction effect we found in the aggregate data is mainly caused by subjects with convex distributional preferences.

The regression shown in Table 5 – based on the subset of data produced by subjects classified as having either convex or linear distributional preferences – confirms this result. We define *peereffect* as 0 when the subject does not change his behavior when he faces a different peer’s choice, 1 if he follows the peer’s choice, and 2 if the change goes against the peer’s choice. Again, we treat treatment NOP as if there were peer information just like in RAF, due to the apparent similarity in observed behavior. The regression results show that while the existence of a peer alone does not explain the peer effect, the cross-term peer*convex (using indicator variable *convex*) does. Thus, only for subjects who are classified as having convex distributional preferences and who face a peer (in RLF and RAF) can we explain the peer effect. We therefore conclude:

**Table 5:** Social interaction effect explained by distributional preferences

*peereffect*	1 (follow peer)			2 (against peer)		
Regressor	Coefficient	(Rob. SE)	$Prob>z$	Coefficient	(Rob. SE)	$Prob>z$
*b1safe*	0.013	(0.063)	0.836	$-$ 0.212	(0.137)	0.121
*peer*	$-$ 0.331	(0.544)	0.544	− 1.676	(1.242)	0.177
*convex*	$-$ 0.869	(0.688)	0.207	$-$ 0.882	(1.339)	0.510
peer*convex	1.517	(0.827)	0.067	2.775	(1.788)	0.221
*cons*	$-$ 0.496	(0.495)	0.316	$-$ 0.873	(0.726)	0.229

Multinomial Logit for *peereffect* (0 is base outcome); N $=$ 132; $Prob > \chi ^{2} = 0.02$, Pseudo $R^{2} = 0.05$

#### **Result 2** (**Distributional Preferences and Social Interaction Effect**)

*In line with Prediction* *2, the social interaction effect observed in the* *aggregate data is mainly caused by subjects with convex distributional* *preferences.*

Our approach in the theory part of the paper was to derive the social interaction effect directly from a DM’s underlying preferences rather than referring to conformism that is not explicitly modeled on the preference level as an explanation. Given Result 2, we conclude that as long as we do not have a good theory about how conformism and convex distributional preferences are related, our approach offers a more direct preference-based explanation of the observed peer effect. Conformity – in the way the term it is used in the economics literature – does not seem to provide a preference-based explanation for why people make the choices we observe.^19^

**Fig. 2:**
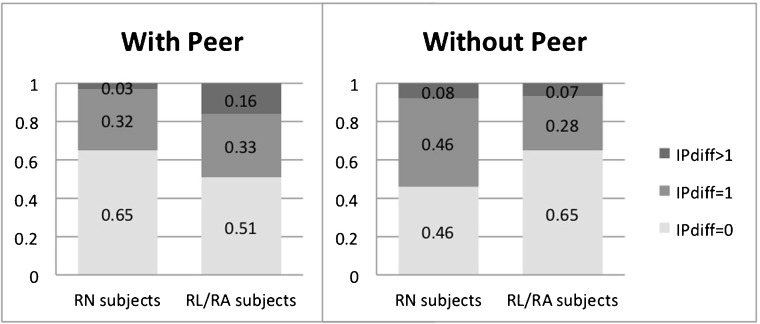
Risk attitude and social interaction effect

In addition to the causal effect of convex distributional preferences on conformistic behavior, our model predicts that the social interaction effect is less pronounced for a risk-neutral DM compared to a risk-loving or risk-averse DM. The left hand side of Fig. 2 shows that in the two treatments with peers (RLF and RAF pooled), about two-thirds of the subjects classified as risk-neutral display no change in the number of safe choices, while for subjects classified as risk-loving or risk-averse, about 50% display changes. Although the difference between those frequencies is quite large, it is not statistically significant (*χ*^2^-test: $p = 0.14$).^20^ One could argue that using *b1safe* to define risk-neutrality may not be appropriate since it allows a subject to behave inconsistently (by switching more than once between the safe and the risky alternative or by switching in the ‘wrong direction’). If, instead, we consider only subjects who switch at most once from the safe to the risky alternative (and never in the other direction), then 30 are classified as risk-neutral, while 68 subjects are risk-loving or risk-averse. Of the risk-neutral subjects, $67\%$ display no change in the number of safe choices, for $33\%$ the point of indifference changes by 1 and no risk-neutral subject displays a change of more than 1. Of the risk-loving or risk-averse subjects, $51\%$ display no change, $33\%$ a change of 1 and $16\%$ a change of more than 1. Comparing those frequencies, we now find significantly different changes in the number of safe choices between consistently deciding subjects who are risk-neutral and consistently deciding subjects who are not risk-neutral (*χ*^2^-test: $p<0.05$) – which is in line with our Prediction 3. In sum, while for our previous definition of risk-neutrality (based on *b1safe*) the results are qualitatively in line with Prediction 3 but statistically insignificant, for the alternative definition based only on consistently deciding subjects, the correlation between risk attitude and change in the number of safe choices is both qualitatively in line with Prediction 3 and statistically significant. For treatment NOP, on the other hand, we observe no such correlations between risk attitudes and change in the number of safe choices, for either definition of risk-neutrality (*χ*^2^-test: $p = 0.44$ in both cases).

#### **Result 3** (**Risk preferences and social interaction effect**)

*Regarding Prediction* *3, we find weak evidence that risk-neutral subjects* *in treatments RLF and RAF have a less pronounced tendency to follow the* *behavior of the peer than risk-loving or risk-averse subjects.*

## Conclusion

The term social interaction effect refers to a particular form of strategic complementarity in which the action choices of agents in a reference group have a positive impact on the DM’s propensity to choose the corresponding action without affecting the DM’s material payoffs. Social interaction effects potentially have important economic consequences, because any change in the environment has not only a direct effect on behavior but also an indirect effect (resulting from the change in the peers’ behavior) of the same sign, and thus a small change in fundamentals might result in a large change in aggregate behavior via the so called “social multiplier”.

We have shown theoretically that convex distributional preferences imply social interaction effects in risky choices, even when the outcomes of a given lottery are stochastically independent across agents deciding for that lottery and even when the DM can only observe the lotteries chosen by the peers but not the corresponding outcomes. Indeed, convex altruistic, inequality averse, maximin, envious, and spiteful preferences all imply that observing (more) peers choose a risky (safe) option increases the DM’s propensity to choose the risky (safe) option as well, although the DM’s material payoffs for the different options remain unaffected by the peers’ choices.

Our experimental results show strong peer group effects in the choices between pairs of lotteries in the sense that observing a peer choose a risky (safe) option increases the DM’s propensity to choose the risky (safe) option as well, although in the experiment the outcomes of a given lottery are stochastically independent across agents and the DM can only observe the lottery chosen by the peer but not the corresponding outcome. Taking advantage of the controlled environment, we have excluded standard identification problems (self-selection, correlated effects, and contextual effects), material payoff externalities and informational externalities as possible explanations and we have concluded that a plausible cause for the observed correlation in risky choices is social interaction effects caused by convex distributional preferences. Support for this conclusion comes from the data analysis on the individual level, which reveals correlations in line with our theory: The social interaction effect observed in the aggregate data is mainly caused by subjects with convex distributional preferences, and the effect seems to be more pronounced for subjects with non-linear risk attitudes than for risk-neutral subjects, although the evidence for this latter comparison is less conclusive.

## Electronic supplementary material

Below is the link to the electronic supplementary material.

(PDF 721 KB)

## Footnotes

1 Part (ii) of our definition is not met if, for instance, there are *informational externalities* such that the DM can learn something about the consequences of a given choice by observing his peers’ behavior – as in the literature on social learning (or herd behavior, or informational cascades; see Gale 1996 for a survey). Or, if there are direct *material-payoff complementarities* implying that an action becomes more profitable for the DM in material terms if others choose the same action – as, for instance, in the models studied in the network externality literature (see Farrell and Saloner 1985, among others). Finally, *correlation of individual characteristics* and *influence of* group characteristics or of *a* *common environment* on behavior might lead to a positive correlation in decisions (see Manski 1993 for a discussion).

2 The defining feature of distributional preferences is that DMs care not only about their own (material) well-being, but also about the (material) well-being of others.

3 Here and throughout the paper *convexity* refers to the shape of upper contour sets. Considering a two person context, denoting the DM’s own material payoff by *m* and the peer’s payoff by *o*, and assuming that the DM’s well-being is strictly increasing in *m*, upper contour sets are to the right of the DM’s indifference curves in (*m*, *o*)-space. Increasing benevolence (or decreasing malevolence) as *o* increases (that is, as one moves southward) along an indifference curve is then equivalent to convexity of upper contour sets (see Cox et al. 2008 for details).

4 While Güth et al. (2008) find that other-regarding concerns are behaviorally relevant when the DM’s own payoff is safe and immediate, but irrelevant when it is risky or delayed, Brennan et al. (2008) find that the risk in the outcome of others is much less important for decision making than the risk in the DM’s own outcome.

5 In the piecewise linear model above, inequality aversion translates to the parameter restriction *min* {− *σ*, $\rho $} $>0,$ with the special case of $-\sigma \geq \rho >0$ for the Fehr and Schmidt model. For an envious DM, we have $-\sigma >\rho = 0$. A DM with maximin preferences, Rawlsian preferences, or Leontief preferences decides as if $\rho >\sigma = 0$. Note that all distributional preference types mentioned up to now fit into the parameter restriction $ \rho >\sigma $. This is not the case with equity aversion (Charness and Rabin 2002), or equality aversion (Hennig-Schmidt 2002), which imply *min* {*σ*,−*ρ* } > 0, and therewith $\rho <\sigma $. See Kerschbamer (2015) for details.

6 Altruistic, surplus maximizing or social welfare preferences translate to the parameter restriction *min* {*σ*, *ρ* } > 0. For DMs who are spiteful, competitive, status-seeking or interested in relative income we have *max* {*σ*, *ρ* } < 0.

7 The procedure relies on minimal assumptions regarding the rationality of a DM. In terms of axioms on preferences the assumptions are ordering (completeness and transitivity) and strict (own-money) monotonicity – see Kerschbamer (2015) for details. In the main text, DMs whose preferences satisfy those two basic axioms are referred to as “rational”.

8 The latter subjects served as peers without being labeled as such in the instructions.

9 We had one treatment without a peer, where these points are not relevant.

10 This design feature makes sure that (i) all subjects are paid for the same decision task; and (ii) if two or more agents decide for the same lottery the realizations are stochastically independent (“individual gambles”).

11 This is due to the fact that this other subject has a passive role in the current round and thus cannot make a new decision; the computer implements his past decision for the current round.

12 Again ordering (completeness and transitivity) and strict monotonicity are the two requirements for rationality. Note that we will not be able to identify a subject’s precise indifference probability (as defined in the theory section), as the experiment features only discrete changes in probabilities. Instead, a lower and upper bound for the indifference probability of a rational subject is identified by the probability *p* in the last pair for which the subject decides for the sure payoff and the probability *p* in the first pair for which the subject decides for the cash gamble.

13 Strictly speaking, the theoretical concept of an indifference probability would require consistent choices, implying at most one switch from the sure payoff to the lottery. Taking the number of safe choices as a proxy for the indifference probability allows us to include all observed choices.

14 By using the two subjects with the most extreme risk attitudes in Block 1 as peers in Block 2 and Block 3 we tried to maximize the number of choices for which the peer in Block 2 made a decision different from the peer in Block 3.

15 One could also compare behavior in Block 1 to behavior in Block 2 or Block 3, but since we did not derive a formal result for a comparison of situations with and without information about the peer’s choice, our main focus will be on the change in behavior from Block 2 to Block 3.

16 Proposition 2 would also predict that subjects with concave distributional preferences have a tendency to deviate from the behavior of the peer. However, since concave distributional preferences are empirically irrelevant, we do not search for evidence in accordance with this prediction in our data.

17 Figure 1 shows that subjects also tend to follow the peer when moving from Block 1 to Block 2. As mentioned earlier, this might be regarded as a comparison between behavior without information about the choice of a peer and behavior with a peer, which is related to – but not identical with – the comparison we are mainly interested in.

18 The fact that the coefficient for *peer* is significant and negative indicates that the choices in Block 2 and Block 3 of treatment NOP are more risk-averse than those of RLF and RAF.

19 Basic motivations that imply conformistic behavior are discussed in the psychology literature – see Cialdini and Goldstein (2004), for instance. None of the discussed motivations, however, predicts a correlation between convex distributional preferences and conformity.

20 The fact that our definition of risk-neutrality may include slightly risk-loving and slightly risk-averse subjects is perfectly consistent with the theory, since in theory the curve for the size of the difference in indifference probabilities as a function of risk attitude is U-shaped with the minimum at risk-neutrality. See Appendix B for details.
