# Supplementary material for: Social interaction effects: The impact of distributional preferences on risky choices
Source: J Risk Uncertain. 2018 May 8;56(2):141–64. doi: 10.1007/s11166-018-9275-5 (PMC5978880; doi:10.1007/s11166-018-9275-5)
Supplement: Supplementary file 1 — (PDF 721 KB) [file 11166_2018_9275_MOESM1_ESM.pdf]

Appendix
Social Interaction Effects: The Impact of Distributional Preferences on Risky Choices

Anita Gantner* and Rudolf Kerschbamer
Department of Economics, University of Innsbruck

published in *Journal of Risk and Uncertainty*

Appendix A:
Extensions within the Basic Model

A.1 More than Two Agents and More than Two Binary Lotteries

For simplicity, we start with $K = 2$ agents and $N \geq 2$ lotteries, and extend our definitions then to more than two agents.

Definition 1. *Suppose that in the* ***two-player case*** *lottery L_n, with $n = 1, 2, ..., N$, yields outcome x_n with probability p_n and outcome zero with probability $1 - p_n$. Let $0 < p_1 < p_2 < ... < p_{N-1} < p_N \leq 1$ and $0 < x_N < x_{N-1} < ... < x_2 < x_1$, i.e. a lottery with a lower index is riskier than a lottery with a higher index. Let u_{ln} denote the expected utility of the DM if he chooses lottery L_l while his peer chooses L_n.*

If the DM has a utility function as given in Equation (1), u_{ln} can be expressed as

$$u_{ln} = p_l x_l (1 - \rho) + p_n x_n \sigma + p_l p_n (\rho - \sigma) \min\{x_l,\ x_n\} \tag{A1}$$

For the **(K > 2) player case**, that is, for a social environment in which there are

*Corresponding author. Address: University of Innsbruck, Department of Economics, Universitätsstrasse 15, A-6020 Innsbruck, Austria. Email: anita.gantner@uibk.ac.at.

$K-1>1$ peers, the Charness and Rabin function extends to:

$$u_{\rho,\sigma}(m,o_1,...,o_{K-1}) = m + \frac{\sigma}{K-1}\sum_{k=1}^{K-1}\max\{o_k - m,\ 0\} - \frac{\rho}{K-1}\sum_{k=1}^{K-1}\max\{m-o_k,\ 0\}, \tag{A2}$$

where $\max\{\rho,\sigma\}<1$ and where o_k is the material payoff of peer $k \in \{1,...,K-1\}$.[1]

Definition 2. *Suppose that in the* ***$K>2$ player case*** *we have $N \geq 2$ lotteries as in Definition 1. Let c_n be the fraction of peers that choose lottery n. Let $u_l(c)$ denote the expected utility of the DM if he chooses lottery L_l while his peers choose according to $c=(c_1,c_2,...,c_{N-1},c_N)$.*

Then $u_l(c)=\sum_{n=1}^{N} c_n u_{ln}$, and using the utility function as given in Equation (A2), we have

$$\begin{aligned} u_l(c) &= p_l x_l(1-\rho) + \sigma\sum_{n=1}^{N} c_n p_n x_n + p_l(\rho-\sigma)\sum_{n=1}^{N} p_n c_n \min\{x_l,x_n\} \qquad \text{(A3)} \\ &= p_l x_l(1-\rho) + \sigma\sum_{n=1}^{N} c_n p_n x_n + p_l(\rho-\sigma)\left(\sum_{n=1}^{l} p_n c_n x_l + \sum_{n=l+1}^{N} p_n c_n x_n\right) \end{aligned}$$

Proposition A1. ***(Distributional Preferences and Risk Attitudes with N Lotteries and K Agents)****. Suppose a DM with a utility function as given in (A2) has to choose between N lotteries which have the same expected value, that is, $\forall n \in \{1,2,...,N\}$ we have $p_n x_n = \kappa$ for some constant κ. Further suppose that the DM observes his peers choose according to $c=(c_1,..,c_{s-1},c_s,c_{s+1},..,c_N)$, where L_s denotes the safest lottery chosen by a strictly positive fraction of the peers. Then the DM's ordering over the N lotteries depends on his distributional preferences. If his distributional preferences are*

(i) convex then $L_N \sim L_{N-1} \sim ... \sim L_s \succ L_{s-1} \succ ... \succ L_1$;

(ii) linear then $L_N \sim L_{N-1} \sim ... \sim L_s \sim L_{s-1} \sim ... \sim L_1$;

(iii) concave then $L_N \sim L_{N-1} \sim ... \sim L_s \prec L_{s-1} \prec ... \prec L_1$.

Proof. First note that regardless of whether the DM has convex, linear, or concave distributional preferences, we have $u_l(c)=u_s(c)$ for $l \geq s$. To see this, note

[1] Here we use the convention that the DM is agent K. Note that dividing through $K-1$ ensures that the relative impact of distributional concerns on the DM's utility payoff is independent of the number of players in his reference group. See Fehr and Schmidt (1999) for a similar normalization.

that $u_s(c) = p_s x_s(1-\rho) + \sigma \sum_{n=1}^{N} c_n p_n x_n + p_s(\rho - \sigma) x_s \sum_{n=1}^{s} p_n c_n$ (when the DM also chooses L_s), and $u_l(c) = p_l x_l(1-\rho) + \sigma \sum_{n=1}^{N} c_n p_n x_n + p_l(\rho-\sigma) x_l \sum_{n=1}^{s} p_n c_n$ (when the DM chooses L_l with $l > s$). Then $u_l(c) - u_s(c) = (p_s x_s - p_l x_l)[1 - \rho + \sum_{n=1}^{s} p_n c_n(\rho - \sigma)] = 0$, since $p_s x_s = p_l x_l$. Next, we show that for $s > l > r$ we have $u_s(c) > u_l(c) > u_r(c)$ if the DM has convex distributional preferences, $u_s(c) = u_l(c) = u_r(c)$ if the DM has linear distributional preferences, and $u_s(c) < u_l(c) < u_r(c)$ if the DM has concave distributional preferences. This is easily seen by noting that

$$
\begin{aligned}
u_s(c) &= p_s x_s(1-\rho) + \sigma \sum_{n=1}^{N} c_n p_n x_n + (\rho - \sigma) \sum_{n=1}^{s} p_n c_n p_s x_s \\
u_l(c) &= p_l x_l(1-\rho) + \sigma \sum_{n=1}^{N} c_n p_n x_n + (\rho - \sigma) \left[\sum_{n=1}^{l} p_n c_n p_l x_l + \sum_{n=l+1}^{s} p_n c_n p_l x_n \right] \\
u_r(c) &= p_r x_r(1-\rho) + \sigma \sum_{n=1}^{N} c_n p_n x_n + (\rho - \sigma) \left[\sum_{n=1}^{r} p_n c_n p_r x_r + \sum_{n=r+1}^{s} p_n c_n p_r x_n \right].
\end{aligned}
$$

Thus, for $s > l$ and $p_n x_n = \kappa, \forall n$ we have

$$
u_s(c) - u_l(c) = (\rho - \sigma) \sum_{n=l+1}^{s} p_n c_n (p_s x_s - p_l x_n).
$$

For a DM with convex (linear; concave) distributional preferences this equation is strictly positive (zero; strictly negative) since $\rho - \sigma > 0$ ($\rho - \sigma = 0$; $\rho - \sigma < 0$) and $p_s x_s = p_n x_n > p_l x_n$ for $p_n > p_l$. The argument for $l > r$ is similar. □

Proposition A1 tells us that in a world with N binary lotteries with equal expected values and K agents, a DM with convex (concave) distributional preferences is risk-averse (risk-loving) when comparing lotteries that are more risky than the safest lottery chosen by a strictly positive fraction of peers, but risk-neutral when comparing lotteries that are less risky.

Proposition A2. ***(Distributional Preferences and Social Interaction Effects with N Lotteries and K Agents)****. Suppose a DM with a utility function as given in Equation (A2) is indifferent between two lotteries L_r and L_s with $r < s$ when he observes that the peers choose according to $c = (c_1, ..., c_r, ..., c_s, ..., c_N)$.*

Then observing at least one of the peers switch from L_s to L_r (L_r to L_s) and no peer switch in the opposite direction implies the following orderings over the two lotteries for the DM: If his distributional preferences are

(i) convex then $L_r \succ L_s$ ($L_s \succ L_r$);

(ii) linear then $L_r \sim L_s$;

(iii) concave then $L_s \succ L_r$ ($L_r \succ L_s$).

Proof. If at least one peer switches from L_s to L_r (L_r to L_s) and none switches in the opposite direction, let this be denoted by $\hat{c} = (\hat{c}_1, ..., \hat{c}_r, ..., \hat{c}_s, ..., \hat{c}_N)$, where $\hat{c}_n = c_n$ for $n \neq r, s$, and $\hat{c}_r > c_r$ ($\hat{c}_r < c_r$, respectively), and $\hat{c}_s = c_s - \hat{c}_r + c_r$. We show that for $\hat{c}_r > c_r$ the difference $[u_r(\hat{c}) - u_s(\hat{c})] - [u_r(c) - u_s(c)]$ is strictly positive for convex, strictly negative for concave, and zero for linear distributional preferences. This is easily seen by noting that

$$\begin{aligned} u_r(c) &= p_r x_r(1-\rho) + \sigma \sum_{n=1}^{N} c_n p_n x_n + p_r(\rho - \sigma) \left[\sum_{n=1}^{r} p_n x_n x_r + \sum_{n=r+1}^{N} p_n c_n x_n \right] \\ u_s(c) &= p_s x_s(1-\rho) + \sigma \sum_{n=1}^{N} c_n p_n x_n + p_s(\rho - \sigma) \left[\sum_{n=1}^{s} p_n x_n x_s + \sum_{n=s+1}^{N} p_n c_n x_n \right]. \end{aligned}$$

Thus, we have

$$\begin{aligned} u_r(\hat{c}) - u_s(\hat{c}) &- u_r(c) - u_s(c) \\ &= (\rho - \sigma)\left[(\hat{c}_r - c_r)(p_r x_r - p_s x_s)p_r + (c_s - \hat{c}_s)p_s x_s(p_s - p_r)\right] \\ &= (\rho - \sigma)(\hat{c}_r - c_r)\left[(p_r x_r - p_s x_s)p_r + (p_s - p_r)p_s x_s\right] \\ &= (\rho - \sigma)(\hat{c}_r - c_r)\left[(p_s - p_r)^2 x_s + p_r^2(x_r - x_s)\right]. \end{aligned}$$

Since for $s > r$ we have $x_r > x_s$ and $p_s > p_r$, the sign of $(\rho - \sigma)$ yields the desired results. □

A.2 Proof for the Remark following Proposition 2 (Common Gambles)

Proof. To see that Proposition 2 extends to the case of common gambles, note that with perfect correlation $u_{rr} = p_r x_r$ and $u_{ss} = p_s x_s$, while u_{rs} and u_{sr} remain as given in (3) and (4). Then, for case (i) of Proposition 2 we have to show that (a) and (b) above hold for common gambles. For (a), we have $u_{rr} = u_{sr} \iff$

$p_r x_r(1-\sigma) = p_s x_s[1-\rho+p_r(\rho-\sigma)]$, which in particular implies that $p_r x_r < p_s x_s$, since $1-\sigma > 1-\rho+p_r(\rho-\sigma)$ for $\rho > \sigma$. Thus, while being risk-neutral with independent gambles when the peer chooses the riskier lottery, a DM with convex distributional preferences is risk-loving with common gambles. For (a) to hold, note that $u_{rs} < u_{ss} \iff p_s x_s[1-(1-p_r)\sigma - p_r\rho] > p_r x_r(1-\rho)$, which is true since the DM was shown to be risk-loving, i.e. $p_r x_r < p_s x_s$ and it was also shown that $1-\sigma-p_r(\rho-\sigma) > 1-\rho$. Finally, for (b) we have $u_{rs} = u_{ss} \iff p_r x_r(1-\rho) = p_s x_s[1-(1-p_r)\sigma - p_r\rho]$, which in particular implies that $p_r x_r > p_s x_s$, since $1-\rho < 1-\sigma-p_r(\rho-\sigma)$ for $\rho > \sigma$. The DM is then risk-averse in his choice between lotteries if the peer chooses the safer lottery, just as in the independent gambles case. To complete the argument for the case of convex distributional preferences, we note that condition $u_{rr} > u_{sr}$ is equivalent to $p_r x_r(1-\sigma) > p_s x_s[1-\rho+p_r(\rho-\sigma)]$, which is satisfied since $1-\sigma > 1-\rho+p_r(\rho-\sigma)$ for $\rho > \sigma$, and $p_r x_r > p_s x_s$ by risk aversion. The argument for the other two cases of Proposition 2 follows similar lines.

□

Appendix B:
Extended Model (Departure from Risk-Neutrality)

Suppose the DM values his own monetary payoff m according to the von-Neumann-Morgenstern utility function $v(m)$ when acting in isolation. In order to avoid imposing unnecessary structure on $v(.)$, we exploit the fact that in the basic model the DM decides between lotteries whose support consists of only three monetary payoffs: x_r, x_s and 0. We can thus normalize the utility associated with two payoffs and use the number representing the utility of the third payoff as an index for the DM's risk attitude. Let $v(m) = m$ for $m \in \{0, x_r\}$ and $v(x_s) = v$. Then for $v < x_s$ the DM is risk-loving, and a larger difference $x_s - v$ means more risk-loving; for $v = x_s$ the agent is risk-neutral; and for $v > x_s$ the agent is risk-averse, and a larger difference $v - x_s$ means more risk-averse. In a social comparison context we assume that the DM is risk-neutral regarding the difference between his own and his peer's payoff, but may display other risk attitudes regarding his own material payoffs, as described above. Then the DM's preferences can be represented by the function

$$u_{\rho,\sigma,v}(m,o) = \begin{cases} v(m) + \sigma(o-m) & for \quad o \geq m \\ v(m) + \rho(o-m) & for \quad o < m \end{cases} \quad \forall\ \sigma < 1, \rho < 1, \tag{A4}$$

where $v(0) = 0$, $v(x_r) = x_r$ and $v(x_s) = v$.[2] To preserve monotonicity of the agent's utility with regard to his own material payoff we impose the following parameter restrictions:[3]

R1: $\forall v < x_s : max\{\sigma, \rho\} < v/x_s$

R2: $\forall v \geq x_s : max\{\sigma, \rho\} < (x_r - v)/(x_r - x_s)$.

Given the functional form from (A4), the DM's expected utility u_{ln} from choosing lottery L_l, with $l = r, s$, while his peer chooses lottery L_n, with $n = r, s$, is

$$u_{rr} = p_r^2 x_r + p_r(1-p_r)(x_r - \rho x_r) + (1-p_r)p_r\sigma x_r; \tag{A5}$$

$$u_{rs} = p_r p_s[x_r - \rho(x_r - x_s)] + p_r(1-p_s)(x_r - \rho x_r) + (1-p_r)p_s\sigma x_s; \tag{A6}$$

$$u_{sr} = p_s p_r[v + \sigma(x_r - x_s)] + p_s(1-p_r)(v - \rho x_s) + (1-p_s)p_r\sigma x_r; \tag{A7}$$

$$u_{ss} = p_s^2 v + p_s(1-p_s)(v - \rho x_s) + (1-p_s)p_s\sigma x_s. \tag{A8}$$

Given that the peer chooses L_r, the DM now prefers L_s over L_r if and only if $u_{sr} > u_{rr}$. Using (A7) and (A5), this simplifies to

$$p_s x_s + \frac{p_s(v - x_s)}{1 + p_r(\rho - \sigma) - \rho} > p_r x_r. \tag{A9}$$

Given that the peer chooses L_s, the DM now prefers L_s over L_r if and only if

[2] There are other plausible ways of extending the functional form (1) to lotteries – see Fudenberg and Levine (2012), or Saito (2013), for instance. We chose this simple form mainly for parsimony and transparency, and to stay as close as possible to our baseline functional form (1). With this functional form, a DM's trade-off between the first component of the utility function, which accounts for his preferences over the own monetary payoff, and the second component, which accounts for social preferences, is distorted by the DM's risk attitude regarding his own payoff, but this distortion remains within narrow bounds, as we keep the highest and lowest possible payoff untransformed.

[3] For an agent who is risk-neutral in isolation, those conditions correspond exactly to our original monotonicity restrictions – that is, to $\sigma < 1$ and $\rho < 1$, or $max\{\sigma, \rho\} < 1$.

$u_{ss} > u_{rs}$. Using (A6) and (A8), this simplifies to:

$$\frac{\rho - \sigma}{1 - \rho} \quad > \quad \frac{p_r x_r}{p_s x_s (p_s - p_r)} - \frac{v - \rho x_s}{x_s (p_s - p_r)(1 - \rho)}. \tag{A10}$$

Conditions (A9) and (A10) extend those found earlier for an agent who is risk-neutral in isolation – it is easy to see that for $v = x_s$ they correspond precisely to our earlier conditions (6) and (7). To identify the impact of a change in the choice of the peer on the incentives of a DM behaving in accordance with the functional form (A4) with preference parameters ρ, σ and v, we calculate – for given values of x_r, x_s and p_s and given choice of the peer – the critical probability p_r^* for which the DM is just indifferent between lotteries L_s and L_r. We shall refer to p_r^* as the DM's "indifference probability". Let $p_r^*(L_r)$ denote the value of the DM's indifference probability when the peer chooses L_r, and $p_r^*(L_s)$ the corresponding value when the peer chooses L_s. From (A9) and (A10) it is immediately seen that for $\rho = \sigma$ we have $p_r^*(L_r) = p_r^*(L_s) = \frac{p_s(v - \rho x_s)}{x_r(1-\rho)}$, implying that the indifference probability of a DM with linear distributional preferences is not affected by the peer's choice. For $\rho \neq \sigma$, condition (A9) yields the following equation:

$$p_r^*(L_r) = \frac{x_r(\rho - 1) + p_s x_s(\rho - \sigma) \pm \sqrt{-4x_r(\rho - \sigma)p_s(\rho x_s - v) + [x_r(1 - \rho) - p_s x_s(\rho - \sigma)]^2}}{2x_r(\rho - \sigma)}. \tag{A11}$$

Thus, for $\rho \neq \sigma$ the value for $p_r^*(L_r)$ is the solution to a quadratic equation, which, in general, is not unique. However, taking the parameter restrictions R1 and R2 for monotonicity of utility in own material payoff into account, it can be shown that only the solution with the positive root is admissible.

Turning to the case where the peer chooses L_s, condition (A10) yields the following equation:

$$p_r^*(L_s) = \frac{p_s(v - \rho x_s) + p_s^2 x_s(\rho - \sigma)}{x_r(1 - \rho) + p_s x_s(\rho - \sigma)}. \tag{A12}$$

To evaluate the impact of a change in the peer's choice on the incentives of a DM with given distributional parameters ρ and σ and given risk attitude v in isolation, we define the difference in indifference probabilities as $d(\rho, \sigma, v) = p_r^*(L_s) - p_r^*(L_r)$.

Then $d(\rho, \sigma, v) > 0$ indicates that if the peer chooses L_s, the DM will require a larger p_r to prefer L_r over L_s, compared to the situation where the peer chooses L_r, i.e., he will choose L_s for a larger range of probabilities. Thus, with $d(\rho, \sigma, v) > 0$ the DM has the tendency to follow the peer, with $d(\rho, \sigma, v) < 0$ the DM has the tendency to deviate from the behavior of the peer, and with $d(\rho, \sigma, v) = 0$ the DM's choice is not affected by the decision of the peer. Furthermore, the absolute value of $d(\rho, \sigma, v)$ is a measure for the size of the impact of a change in the peer's choice on the DM's indifference probability.

In the proof below, we show that for $\rho > \sigma$ the difference in indifference probabilities $d(\rho, \sigma, v)$ is positive for each admissible value of v; furthermore, $d(\rho, \sigma, v)$ is convex and it obtains its (unique) minimum at $v = x_s$. By contrast, for $\rho < \sigma$ the difference in indifference probabilities is negative for each admissible value of v; furthermore, $d(\rho, \sigma, v)$ is concave in this case and it obtains its (unique) maximum at $v = x_s$. Together with the observation that for $\rho = \sigma$ we have $p_r^*(L_s) = p_r^*(L_r)$, these findings imply the following result:

Proposition A3. ***(Distributional Preferences and Social Interaction Effect with General Risk Attitudes in Isolation)*** *Suppose a DM whose preferences can be represented by a utility function as in Eq. (A4) is indifferent between lotteries L_r and L_s when he observes the peer choose L_s (L_r). Then observing the peer choose L_r (L_s) instead implies the following orderings over the two lotteries for the DM: If his distributional preferences are*

(i) convex then $L_r \succ L_s$ ($L_s \succ L_r$);

(ii) linear then $L_r \sim L_s$;

(iii) concave then $L_s \succ L_r$ ($L_r \succ L_s$).

Furthermore, for given distributional preference parameters ρ and σ, the size of the impact of a change in the peer's choice on the DM's indifference probability is smaller for a DM who is risk-neutral in isolation than for a DM who is risk-averse or risk-loving in isolation.

Proof. To show that for $\rho > \sigma$ the difference in indifference probabilities $d(\rho, \sigma, v)$ is positive for each admissible value of v, we first take the derivative of $d(\rho, \sigma, v)$ with respect to v. We find the derivative to be

$$\frac{\partial d(\rho,\sigma,v)}{\partial v} = \frac{p_s}{x_r(1-\rho)+p_s x_s(1-\sigma)} - \frac{p_s}{\sqrt{4x_r(\sigma-\rho)(p_s\rho x_s - p_s v) + (x_r - \rho x_r - p_s\rho x_s + p_s\sigma x_s)^2}} \tag{A13}$$

Setting this derivative equal to zero, we find a unique solution at $v = x_s$. Now we just need to evaluate $d(\rho,\sigma,v)$ at $v = x_s$. If its value is positive, we know that $d(\rho,\sigma,v)$ has a minimum at $v = x_s$, and thus $d(\rho,\sigma,v)$ is always positive. Evaluating $d(\rho,\sigma,v)$ at $v = x_s$, we get

$$d(\rho,\sigma,v=x_s) = \frac{2p_s^2\rho^2 x_r x_s - 4p_s^2\rho\sigma x_r x_s + 2p_s^2\sigma^2 x_r x_s - 2p_s^2\rho^2 x_s^2 + 4p_s^2\rho\sigma x_s^2 - 2p_s^2\sigma^2 x_s^2}{2x_r(\rho-\sigma)[x_r(1-\rho)+p_s x_s(\rho-\sigma)]} \tag{A14}$$

Inspection of the numerator in the above equation shows that it can be rewritten as $2p_s^2 x_s(x_r - x_s)(\rho-\sigma)^2$, which is positive for any ρ, σ. Inspection of the denominator shows that if $\rho > \sigma$, then the expression in the denominator is positive. Thus, for $\rho > \sigma$, the term $d(\rho,\sigma,v)$ is positive. If, instead, $\rho < \sigma$, then the first term in the denominator is negative, and for the second term (in brackets) it must be that $\rho - \sigma < 1 - \sigma < 1 - \rho$ since $\rho < 1$. Then $x_r(1-\rho) > p_s x_s(\rho-\sigma)$, since $x_r > p_s x_s$ and $1-\rho > \rho-\sigma$. In this case, the denominator is negative and thus, for $\rho < \sigma$, the term $d(\rho,\sigma,v)$ is negative.

□

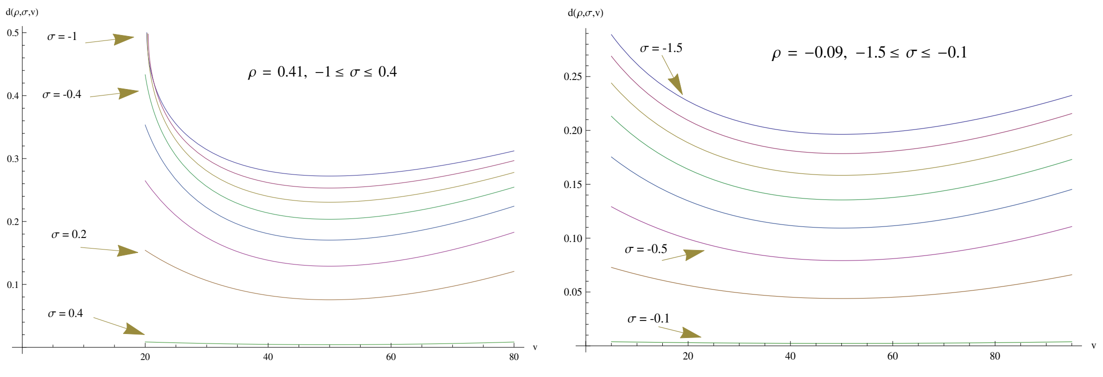

Figure A1: Difference in Indifference Probabilities of DM with Convex Distributional Preferences

Figure A1 illustrates the results for convex distributional preferences using the lottery parameters of the experiment.[4] Figure A1 displays the shape of $d(\rho, \sigma, v)$ for various types of convex distributional preferences. For each curve, ρ and σ are kept fixed and $d(\rho, \sigma, v)$ is plotted as a function of v, where we allow for arbitrary values of v within the admissible range. On the left graph of Figure A1, we fix ρ at some positive value and vary σ, allowing for positive values (such parameter combinations correspond to convex altruism) as well as negative values (such parameter combinations correspond to inequality aversion à la Fehr and Schmidt 1999). On the right graph we fix ρ at some negative value and vary σ, allowing only for values $\sigma < \rho$ to ensure convexity (such parameter combinations correspond to spiteful preferences). Each curve shows the change in the DM's indifference probability when the peer's decision moves from risky to safe. Note first that for all cases of convex distributional preferences, the level of the curve $d(\rho, \sigma, v)$ is positive, i.e. the DM's indifference probability is larger if the peer chooses the safe alternative, and smaller if the peer chooses the risky alternative. In other words, for a risky choice of the peer the DM requires a smaller probability for the favorable outcome x_r to occur in order to prefer the risky alternative. The DM thus follows the peer's behavior. Note further that, as stated in Proposition A3, the U-shaped curves all have their minimum for a risk-neutral DM, i.e. the social interaction effect is smallest here, while it is larger the more risk-averse or risk-loving a DM is.

[4] Remember that in the experiment subjects are exposed to choices where the safer lottery contains no risk at all. Here it is important to note that all our results hold in particular for $p_s = 1$, and we prefer to use a secure payoff in the experiment since it makes the decision less complex for subjects.

Appendix C:
Screenshot of Experiment

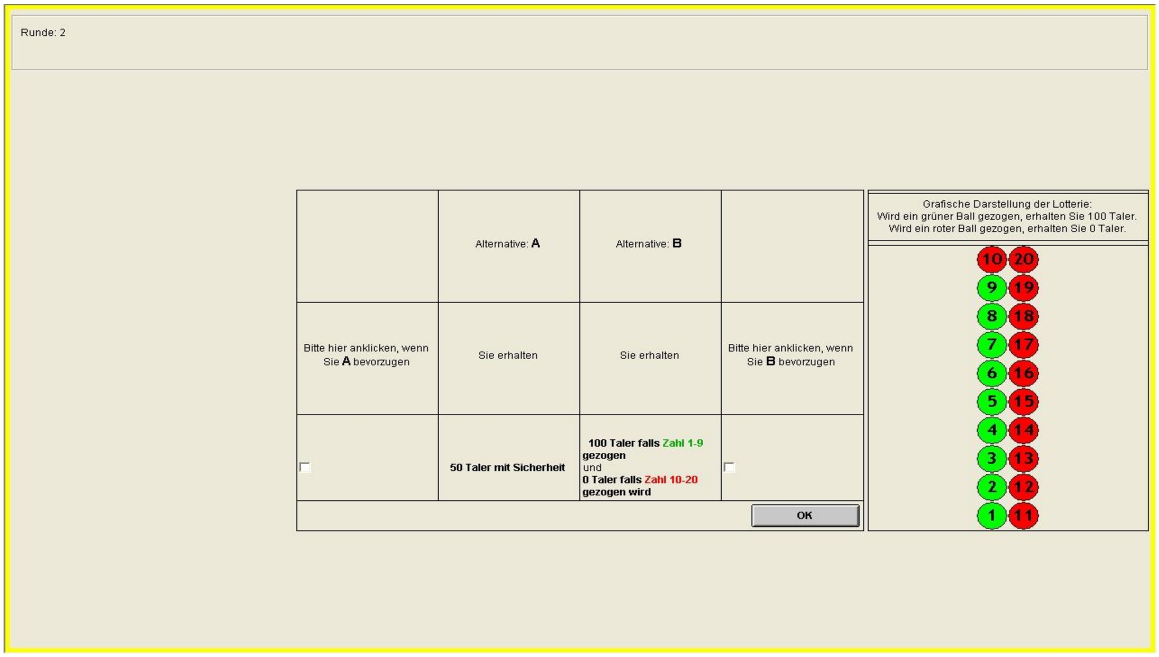

Translation:

	Alternative **A**	Alternative **B**		Graphical representation of the lottery: If a green ball is drawn, you receive 100 Taler; if a red ball is drawn, you receive 0 Taler.
Please click here, if you prefer **A**	You receive	You receive	Please click here, if you prefer **B**	
☐	**50 Taler with certainty**	**100 Taler if number 1-9 is drawn** and **0 Taler if number 10-20 is drawn**	☐	
			OK	

Figure A2: Presentation of a Decision Pair in Part 2 of the Experiment

Appendix D: Experimental Instructions

General Information

You are participating in two experiments on decision making. A research foundation has provided the funds for conducting the experiment. You can earn a considerable amount of money by participating. The text below describes exactly how your total earnings will be determined. For better comprehension only male pronouns are used; they should be understood as gender neutral.

Anonymity:
Your decisions remain anonymous. Neither the experimenters nor other subjects will be able to link you to any of your decisions. In order to keep your decisions private, please do not reveal your choices to any other participant. Each participant will only be informed about his own earnings, but not about the earnings of other participants.

Procedure:
First, Experiment 1 will be described and completed, and then we proceed to Experiment 2. At the beginning of each experiment you will receive precise instructions. We will read the instructions aloud and you will have time for questions. Please do not hesitate to ask questions if there is need for clarification. Upon completion of both experiments you will be paid out your total earnings.

Experimental Currency:
You earnings will be given in **Talers** in both experiments. At the end of both experiments, Talers will be converted into Euros, and you will be paid the Euro amount in cash. A Taler corresponds to 10 Cents, that is

10 Talers correspond to 1 Euro.

No Private Communication:
Please do not talk to other participants. If you have questions after reading the instructions or during the experiment, please raise your hand and one of the experimenters will come to clarify your question.

Experiment 1

In Experiment 1 another participant is assigned to you as your passive partner. **Your decisions** in this experiment have **consequences for you own earnings as well as for the earnings of your passive partner.** Your passive partner, however, cannot affect your earnings. A random decision of the computer will determine who your passive partner is, and you will not know his identity at any point in time. Your passive partner will also never be informed about your identity.

Decisions and Pairs of Alternatives: You will make a total of **10 decisions** in Experiment 1. Each of your decisions is a choice between alternative LEFT and alternative RIGHT. Each alternative is a distribution of Talers between you and your passive partner.

Example: You may be asked whether you prefer alternative LEFT, in which you receive 15 Talers and your passive partner receives 30 Talers, or alternative RIGHT, in which you

receive 20 Talers and your passive partner also 20 Talers. You have to make a choice between these two alternatives. This decision problem would be represented in the following way:

	Alternative: **LEFT**		Alternative: **RIGHT**		
Please click here if you prefer **LEFT**	*you receive*	*passive partner receives*	*you receive*	*passive partner receives*	*Please click here if you prefer* **RIGHT**
□	15 Talers	30 Talers	20 Talers	20 Talers	□

Your earnings from Experiment 1 will be determined in the following way:

Earnings as active participant: At the end of the experiment **one** of the 10 decision problems is selected by a random draw made separately for each participant, and the alternative chosen in this decision problem is paid out in real money. Each **random draw** is made publicly **from a bingo cage with ten numbered balls** (with numbers from 1-10). All numbers are equally likely to be drawn. The number on the ball drawn for you determines the decision problem from which you receive your payoff as active participant. This number is entered in the computer, and the computer assigns the corresponding payoff to you. For example, if the decision problem above were chosen for you, and if you had chosen alternative LEFT, then you would receive 15 Talers as active participant, while your passive partner would receive 30 Talers from his role as a passive partner.

Earnings as passive partner: Just like your passive partner receives money from your decision without doing anything, you receive money from another participant without doing anything, i.e. you are the passive partner of some other participant. The computer program ensures that you are not assigned the same person as active participant and passive partner. That is, if your decision determines the payoff of participant x, then the decision of participant x will not determine your payoff, but that of another participant.

To summarize: In Experiment 1 you will make 10 decisions, one of which will determine your actual payoff as active participant. At the time when you make your decisions, you will not know which of the 10 decisions will determine your payoff from the role of an active participant. All of your decisions are equally likely to be drawn for determining the payoff. In addition to your earnings in the role of an active participant you will also receive earnings from the role of a passive partner. You cannot affect the amount of your earnings in the role of a passive partner; it depends exclusively on the decisions of another participant. **The computer program ensures that you are assigned different partners in both roles.**

The draws for determining the earnings from Experiment 1 will be made after Experiment 2 is completed.

Experiment 2

Your decisions in Experiment 2 have **consequences for your own earnings only; they do not affect the earnings of other participants**. This is also true for all other participants: they can only affect their own payoffs but not the payoffs of any other participant.

Decision rounds and pairs of alternatives: Experiment 2 consists of 30 decision rounds. In each round, you will see a pair of alternatives on the screen. All participants see the same pair of alternatives in each round. Each pair of alternatives consists of Alternative A and Alternative B. **Alternative A is always a sure payoff, Alternative B is always a lottery**.

Active and passive role: In some rounds, you will see pairs of alternatives which you have already seen in previous rounds. In such rounds, some participants have an active role, and others have a passive role. Participants in an active role have to make a new decision. Participants in a passive role do not make a new decision; the computer automatically implements the decision they made the first time they saw this pair of alternatives. In rounds with active and passive roles you will be informed at the beginning of a round which role is assigned to you.

Information: When you see a pair of alternatives for which you have already made a decision in a previous round, the screen will display which decision you made the first time you saw this pair of alternatives. If you are in an active role, you will also be informed about the decision some other participant made the first time he saw this pair of alternatives. This information will be displayed on your screen. The other participant is now in the passive role, that is, the computer automatically implements the same decision that he made the first time he saw this pair of alternatives. Thus, **you know precisely how this other participant decides in the current round**. This other participant remains anonymous for you, that is, you will not get to know his identity at any point in time. As mentioned above, your earnings in Experiment 2 do not depend on the decisions of other participants, but only on your own decisions.

Your task in a decision round: If you are in an active role in a given round, you will be asked to make a decision between the two alternatives. You may change your decision as long as you have not clicked the "confirm" button. If you click "confirm", you will go to the next round with a new pair of alternatives. If you are in a passive role in a given round, the computer will implement your previous decision for this pair of alternatives. You are only asked to click "confirm" in order to get to the next round.

Your earnings from Experiment 2 will be determined at the end by using the following two-stage procedure:

Stage 1: From a **bingo cage with 30 numbered balls** (numbers from 1-30) **a ball will be drawn**, visible to anyone in this room. All balls are equally likely to be drawn. The number on the drawn ball determines the round that will be paid out. Therefore**, the round that determines earnings from Experiment 2 is the same for all participants.**

Stage 2: A separate random draw follows for each participant. The bingo cage now contains 20 numbered balls (numbers from 1-20) for each draw. Again, all balls are equally likely to be drawn. The number on the ball is the **personal lucky number** of the respective participant. If this participant has chosen Alternative A in the round that is determined to be

paid out (in Stage 1), then he receives the corresponding sure payoff independent of his personal lucky number (since Alternative A is always a sure payoff). If, instead, the participant has chosen Alternative B in that round, then his personal lucky number determines his payoff as shown in the following example:

Example: Suppose that in Stage 1 the number 7 is drawn. This means that for all participants round 7 will be paid out. Suppose that in this round the following pair of alternatives was presented:

	Alternative **A**	Alternative: B	
Please click here if you prefer **A**	*you receive*	*you receive*	*Please click here if you prefer* **B**
□	**50 Talers**	**100 Talers if number 1-6 drawn**, and **0 Talers if number 7-20 drawn**	□

Your earnings from Experiment 2 depend on how you decided in this round. If you chose **Alternative A**, then you receive a total of 50 Talers from Experiment 2 independent of your personal lucky number. If you chose **Alternative B**, then your earnings from Experiment 2 depend on your personal lucky number. Suppose that in Stage 2 the number 3 was drawn as your personal lucky number. In this case you receive 100 Talers if you chose Alternative B, since your personal lucky number is between 1 and 6. Suppose another participant also chose Alternative B in this round, but the number 8 was drawn as his personal lucky number. Then he receives 0 Talers.
